# A new era of efficiency: artificial intelligence scribes and the future of ophthalmology

**DOI:** 10.1038/s41433-026-04346-y

**Published:** 2026-02-18

**Authors:** Amy Wang, Victor F. A. Almeida

**Affiliations:** 1 Case Western Reserve University School of Medicine, Cleveland, USA; 2 Department of Anesthesiology, Cleveland Clinic Foundation, Cleveland, USA

**Keywords:** Health services, Health occupations

Effective medical documentation in the Electronic Health Record (EHR) is essential for enhanced communication, quality longitudinal patient care, patient safety, legal protection, and billing. However, extensive amounts of time spent on EHR documentation increases clerical workload, which has been associated with decreased direct physician-patient interaction and a decline in physician overall well-being [1–5]. New artificial intelligence (AI) technology is rapidly being adapted for medical documentation in an effort to address this problem. Overall physician usage of AI nearly doubled from 2023 to 2024 from 38% to 66%, with the most prevalent use in documentation [6]. Ophthalmologists in particular may benefit from the use of AI scribes due to the fast paced physician-patient interactions, highly detail-oriented clinical and surgical encounters, and the need to document large volumes of data, including clinical notes and imaging results. However, the current literature is lacking with regards to AI scribing in this specialty, emphasising the need for increased discussion and further research into the safe and effective integration of AI scribing tools within ophthalmology practices.

In ophthalmology, AI-assisted documentation functions through a multi-step process. First, AI scribes capture physician-patient interactions in real time and automatic speech recognition technology converts speech to text, allowing for hands-free recording of the visit. The recorded patient encounter can be combined with other structured data such as visual acuity and intraocular pressure measurements through natural language processing algorithms to extract key clinical findings [7]. Deep learning models can also be used for analysis of images such as fundus and retinal imaging and optical coherence tomography [8]. Second, large language models (LLMs) trained on medical data synthesise and organise the data to draft ophthalmology-specific clinical notes, including subjective, objective, assessment, and plan sections. They can also create patient instructions and after-visit summaries, which are generated within minutes of the end of the appointment [9]. Lastly, the clinical note is presented to the ophthalmologist for review of accuracy and completeness, where it is then entered into the EHR. Certain AI scribing tools (Nuance DAX Copilot by Microsoft) are already directly integrated into EHRs such as Epic, enabling notes to be directly inserted into the patient chart after being reviewed by the physician, facilitating ease of use and minimal disruption to clinical workflow [10].

The most consistently observed advantage of AI scribes within the clinic is a reduction in time spent on EHR documentation per appointment and after hours [1, 11, 12]. In a fast-paced, high patient volume field such as ophthalmology, the need for detailed documentation for each clinical encounter creates an ideal opportunity for the utilisation of AI scribes for more efficient documentation. Additionally, hands-free note taking and decreased time in the EHR allows ophthalmologists to dedicate more attention to patients during encounters. Physicians are able to spend more time on non-documentation activities, such as increased face-to-face interaction with patients [1]. Physicians also report increased opportunities to build better relationships with patients by providing more direct eye contact instead of dividing their attention between the patient and computer [13, 14]. As a result, AI scribes in ophthalmology allow for more productive clinic flow in a busy clinical practice and increased quality of patient-physician interactions within a short patient encounter.

In addition to applications in the clinic, automated transcription with AI scribing can also be utilised in the operating room. Ophthalmologic surgery is a highly precise and delicate form of microsurgery, which requires clear communication between a multidisciplinary surgical team. AI scribes within the operating room can capture intraoperative dialogue, documenting surgical steps and possible surgical complications such as posterior capsule rupture or intraocular haemorrhage in real time. This facilitates accurate documentation of intraoperative decision-making to optimise patient outcomes, while maintaining seamless flow of care.

AI scribe models are also able to generate patient-facing summary notes in easy to understand language, with aims at targeting a 6th–7th grade reading level as recommended by the National Institutes of Health [15]. The ability to adjust terminology based on the patient’s health literacy level is particularly relevant in ophthalmology, where technical jargon and abbreviations are common and many clinical concepts are unfamiliar to the general population. As a result, patients frequently struggle to understand what was done during the visit and what the next steps in management are. Patient-friendly notes that use plain language can improve readability and enhance understanding of eye conditions [16], which could therefore improve comprehension, patient engagement, and treatment adherence post-clinic and post-surgery.

The most prominent limitation of AI scribes is the risk for error in documentation and voice recognition, and its inability to assess body language and other unspoken signs that can be picked up by humans in a face-to-face exchange [17–19]. This potential for error increases the risk for documentation gaps and inaccurate clinical records. Additionally, the “blackbox” nature of many AI models remains a concern, as the training data and decision-making processes underlying model outputs are often not visible to clinicians, raising the possibility of unintended biases. Such biases can limit the generalisability of AI scribing algorithms and perpetuate health disparities in underserved or minority populations [20]. Finally, AI scribes transcribe sensitive patient information, which raise concerns for data privacy and security such as data breaches, unauthorised access, and misuse. These concerns emphasise the need for data protection, patient consent and transparency in the mechanisms underlying AI models [19, 21, 22].

Artificial intelligence-based transcription tools mark a major advancement in modern ophthalmology, with uses in clinical care and operating room procedures. AI scribing increases ophthalmology workflow efficiency, quality of patient interactions, and patient comprehension in a high patient volume specialty. However, their use entails many limitations that necessitate strict physician oversight and robust privacy protections. This rapidly growing technology will transform ophthalmic medical documentation in the future, highlighting the need for increased discussion and further research into the safe and effective integration of AI scribing tools within ophthalmology.
